# Supplementary material for: Improving polygenic prediction from summary data by learning patterns of effect sharing across multiple phenotypes
Source: bioRxiv. 2024 May 10:2024.05.06.592745. Preprint. [Version 1] doi: 10.1101/2024.05.06.592745 (PMC11100663; doi:10.1101/2024.05.06.592745)
Supplement: Supplement 2 [file media-2.pdf]

Table 1: **Mean prediction R^2 across folds for the 16 blood cell traits in the full and sampled UK Biobank data.**

**Phenotype**	**Full data**			**Sampled data**		
	LDpred2-auto	*SBayesR*	*mr.mash-rss*	*LDpred2-auto*	*SBayesR*	*mr.mash-rss*
Red Blood Cell Counts (RBC#)	0.1554	0.1577	**0.1594**	0.0883	0.0983	**0.1043**
Haemoglobin Concentration (HGB)	0.1162	**0.1182**	0.1164	0.0622	**0.0711**	0.0706
Mean Corpuscular Volume (MCV)	0.2273	0.2294	**0.2325**	0.1624	0.1727	**0.1750**
Red Blood Cell Volume Distribution Width (RDW)	0.1612	0.1635	**0.1689**	0.1028	0.1144	**0.1230**
Mean Sphered Cell Volume (MSCV)	0.1738	0.1765	**0.1843**	0.1129	0.1229	**0.1322**
Reticulocyte Percentage (RET%)	0.1366	0.1403	**0.1452**	0.0893	0.0991	**0.1073**
High Light Scatter Reticulocytes Percentage (HLR%)	0.1445	0.1482	**0.1502**	0.0959	0.1065	**0.1115**
Platelet Count (PLT#)	0.2385	0.2406	**0.2428**	0.1595	0.1702	**0.1775**
Plateletcrit (PCT)	0.1961	0.1982	**0.1993**	0.1213	0.1307	**0.1346**
Platelet Distribution Width (PDW)	0.2159	0.2182	**0.2196**	0.1524	0.1633	**0.1661**
White Blood Cell Count (WBC#)	0.1120	0.1135	**0.1155**	0.0564	0.0640	**0.0667**
Lymphocyte Percentage (LYMPH%)	0.0901	0.0917	**0.0964**	0.0422	0.0503	**0.0561**
Monocyte Percentage (MONO%)	0.1594	0.1624	**0.1644**	0.1081	**0.1175**	0.1159
Neutrophil Percentage (NEUT%)	0.0837	0.0858	**0.0918**	0.0402	0.0480	**0.0546**
Eosinophil Percentage (EO%)	0.1348	**0.1365**	0.1339	0.0714	**0.0822**	0.0802
Basophil Percentage (BASO%)	0.0213	0.0249	**0.0283**	0.0073	0.0143	**0.0162**

Highlighted in **bold** is the highest R^2 value across methods for each trait.
